# PD-L1 Deficiency within Islets Reduces Allograft Survival in Mice

**DOI:** 10.1371/journal.pone.0152087

**Published:** 2016-03-18

**Authors:** Dongxia Ma, Wu Duan, Yakun Li, Zhimin Wang, Shanglin Li, Nianqiao Gong, Gang Chen, Zhishui Chen, Chidan Wan, Jun Yang

**Affiliations:** 1 Institute of Organ Transplantation, Tongji Hospital, Tongji Medical College, Huazhong University of Science and Technology, Key Laboratory of Organ Transplantation, Ministry of Education, Key Laboratory of Organ Transplantation, Ministry of Health, Wuhan, Hubei Province, P. R. China; 2 Department of Endocrinology, Tongji Hospital, Tongji Medical College, Huazhong University of Science and Technology, Wuhan, Hubei Province, P. R. China; 3 Department of Hepatobiliary Surgery, Union Hospital, Tongji Medical College, Huazhong University of Science and Technology, Wuhan, Hubei Province, P. R. China; Children's Hospital Boston/Harvard Medical School, UNITED STATES

## Abstract

**Background:**

Islet transplantation may potentially cure type 1 diabetes mellitus (T1DM). However, immune rejection, especially that induced by the alloreactive T-cell response, remains a restraining factor for the long-term survival of grafted islets. Programmed death ligand-1 (PD-L1) is a negative costimulatory molecule. PD-L1 deficiency within the donor heart accelerates allograft rejection. Here, we investigate whether PD-L1 deficiency in donor islets reduces allograft survival time.

**Methods:**

Glucose Stimulation Assays were performed to evaluate whether PD-L1 deficiency has detrimental effects on islet function. Islets isolated from PDL1-deficient mice or wild- type (WT) mice (C57BL/6j) were implanted beneath the renal capsule of streptozotocin (STZ)-induced diabetic BALB/c mice. Blood glucose levels and graft survival time after transplantation were monitored. Moreover, we analyzed the residual islets, infiltrating immune cells and alloreactive cells from the recipients.

**Results:**

PD-L1 deficiency within islets does not affect islet function. However, islet PD-L1 deficiency increased allograft rejection and was associated with enhanced inflammatory cell infiltration and recipient T-cell alloreactivity.

**Conclusions:**

This is the first report to demonstrate that PD-L1 deficiency accelerated islet allograft rejection and regulated recipient alloimmune responses.

## Introduction

Currently, islet transplantation is becoming one of the most promising remedies for T1DM, an insulin-dependent diabetes mellitus, because it is safer, more effective and less invasive. Many factors influence islet graft survival and function. Some factors are intrinsic to the graft itself, and others originate from the recipient [1–3]. Immunologic rejection is one of the most important causes contributing to allograft failure. In recent years, many immunosuppressive agents have been tested with the goal of reducing rejection during transplantation procedures. However, there are many side effects to this approach, such as infection and nephrotoxicity [4, 5]. Thus, many approaches are being tested for use during islet transplantation to improve allograft survival without using any immunosuppressive agents [6–9].

Alloreactive T cell activation greatly affects allograft rejection, which is controlled by both signals 1 and 2. With signal 2 and costimulatory pathways, many negative signals inhibit T cell activation and terminate immune responses [10, 11]. PD-L1 is a recently identified negative costimulatory molecule in the CD28–B7 family. PD-L1 is expressed in many immune cells and non-hematopoietic tissues. PD-1, the PD-L1 receptor, is expressed on activated T, B, and myeloid cells [12, 13]. The PD-1:PD-L1 interaction regulates alloimmune responses [14]. Paolo Fiorina[15] reported that autologous HSCs can be mobilized with a CXCR4 antagonist to promote the long-term survival of islet allografts, an effect that is partly dependent on the PD-L1 mechanism. In addition, the local overexpression of PD-L1 in mouse islets prolongs allograft survival. Furthermore, Yang et al reported that PDL1-deficient donor hearts significantly accelerated cardiac allograft rejection and vasculopathy using a heart chronic rejection model in mice. However, PDL1- deficient recipients did not exhibit an accelerated cardiac allograft rejection [16]. Thus far, little is known about the role of PD-L1 deficiency within the donor islets and how this deficiency regulates allograft rejection.

Here, we studied PD-L1 deficiency in donor islets and investigated alloimmune response regulation *in vivo*. Islets from PD-L1 deficient mice or wild type (WT) mice (C57BL/6j) were isolated and transplanted beneath the renal capsule of diabetic BALB/c mice to determine whether PD-L1 deficiency reduces the survival time of pancreatic islet allografts. We also explored the possible mechanisms.

## Materials and Methods

### Mice

Inbred male BALB/c (H-2d), C57BL/6 (B6; H-2b, WT) mice were obtained from the Beijing HFK Bioscience CO., LTD (Beijing, China). PD-L1^-/-^mice were originally purchased from the Jackson Laboratory (Bar Harbor, ME, USA). The mice were bred and housed under specific pathogen-free conditions at the Tongji Medical School Facilities for Animal Care and Housing. Mice were between 6 and 8 weeks of age at the time of the first procedure. All procedures involving animal use in this study were performed and monitored in accordance with the guidelines of the Chinese Council on Animal Care and approved by the Institutional Animal Care and Use Committee of the Tongji Medical College, Huazhong University of Science and Technology (Permit Number:TJ-A20121211). All surgeries were performed under anesthesia with sodium pentobarbital, and all animal suffering were minimized.

### Induction of diabetes

Streptozotocin (STZ) (Sigma, MO, USA) was dissolved to a 10g/L solution (pH = 4.4) with citric acid and sodium citrate buffer. BALB/c mice were fasted for 20 hours (without water deprivation) and then were given STZ (120 mg/kg) via a single abdominal injection. Tail vein blood was obtained 48 hours later to determine blood glucose levels. Animals with persistent random blood glucose levels >16.7 mmol/L were regarded as diabetic animal models and were the transplant recipients.

### Islet isolation and purification

Islets were isolated from the pancreas of PD-L1^-/-^ and WT mice according to the modified classic method of collagenase type V (1.0 mg/ml, Sigma, MO, USA) infusion via injection into the common bile duct, vigorous agitation, discontinuous Ficoll gradient centrifugation [17], and purification by hand selection with a microscope.

### Islet transplantation

Recipient diabetic BALB/c mice fasted for 12 hours before transplantation. According to Gotoh et al [18], 400 islets from WT or PD-L1^-/-^ mice were transplanted into the renal subcapsular space of the recipient to establish stable models of mouse pancreatic islet transplantation. Normal primary function of grafted pancreatic islets was defined as a blood glucose concentration <11.11 mmol/L 3 days after transplantation. Then, we monitored blood glucose changes and the postoperative survival time of grafted pancreatic islets at different time points. Pancreatic islet rejection was defined as a blood glucose concentration >14.5 mmol/L for at least two consecutive days after successful transplantation. The first day was defined as the time of the acute rejection reaction. The survival time of grafted pancreatic islets was the number of days from pancreatic islet transplantation to acute rejection.

### Glucose stimulation assay

According to Wang et al [19] and Naoaki Sakata et al [20], fresh islets isolated from WT or PD-L1^-/-^ mice were cultured in Krebs-Ringer’s Buffer (Sigma, MO, USA) at 37°C for 30 min. Then, the supernatant was replaced with Krebs-Ringer’s Buffer containing 5 mmol/L or 20 mmol/L glucose. After incubating the culture for 60 min in 37°C, the supernatant was collected and stored at -80°C for insulin measurement by a sensitive insulin enzyme-linked immunosorbent assay (Crystal Chem., Chicago, IL, USA). The stimulation index was calculated by dividing the insulin release following high glucose by the insulin release following low glucose.

### Immunofluorescence and immunohistochemistry

The part of the kidney bearing grafted islets was fixed in 4% paraformaldehyde, paraffin embedded, and stained with hematoxylin and eosin (H&E). To visualize the islets transplanted beneath the kidney capsule, the sections were stained with rat anti-insulin. To detect inflammatory cell infiltration, we applied rat anti-CD4 CD8, and Foxp3 (BD Bioscience, Franklin Lakes, NJ, USA) antibodies, followed by secondary Texas Red or HRP—conjugated antibodies. Nuclear counterstaining was performed using 4,6-diamidino-2-phenylindole (DAPI), and the sections were viewed with a Nikon Eclipse Ti-SR epifluorescence microscope (Nikon, Tokyo, Japan).

### Flow cytometry

Splenocytes were obtained from recipients 5 days after transplantation and stained with fluorochrome-labeled monoclonal antibodies (mAbs) against CD4, CD8, CD62 ligand (CD62 L), and CD44 (eBioscience, San Diego, CA, USA). For the intracellular staining of foxp3, splenocytes were fixed and permeabilized with Fixation/Permeabilization buffer and then stained with an anti-foxp3 antibody (eBioscience). For cytokine staining (IFN-γ, IL-4 and IL-17), the cells were first incubated with 1x cell stimulation cocktail (eBioscience) for 4 h. The cells were then fixed and permeabilized with Fixation/Permeabilization buffer (eBioscience), followed by staining with fluorochrome-labeled mAbs against IFN-γ, IL-4, and IL-17 and an isotype control. Flow cytometry analysis was performed with a FACSCaliber system (BD Biosciences), and analyzed with FlowJo software.

### Mixed-lymphocyte reaction

Splenocytes from recipient mice were harvested 5 days after transplantation and were termed responders. In total, 0.5x10^6^ responder cells were cultured with equally irradiated (35 Gy) stimulator cells from the donor spleen. IFN-γ was quantified after the cells were cultured for 36 h. IL-4 and IL-17 were quantified after 4 days of culture. All of the cytokines were detected using Flow Cytometry

### ELISpot Assay

The ELISpot assay (Dakewe Bioscience Co., Ltd., Beijing, China) described by Yang et al[16] was adapted to measure the frequency of alloreactive T cells producing interferon-γ (IFN-γ). Splenocytes from recipient mice were harvested 5 days after transplantation and used as responders. Splenocytes obtained from donor mice were used as stimulators. A total of 0.2x10^6^ responder cells were cultured with an equal number of irradiated (35 Gy) stimulator cells for 24 h. The frequencies of cells secreting IFN-γ were the number of spots containing 0.2x10^6^ splenocytes that produced this cytokine.

### Statistics

Data, which are expressed as the means±SD, were obtained from at least three individual experiments, and statistical comparisons were performed using Student’s t tests. Allograft survival curves were created by Prism 6.01 software using Kaplan-Meier life-table analyses, and the survival differences between groups were compared using the log-rank test (GraphPad Prism 6.01). Differences were considered significant if P<0.05.

## Results

### PD-L1 deficiency exhibited no pernicious effects on islet β cell function *in vitro*

**Fig 1:**
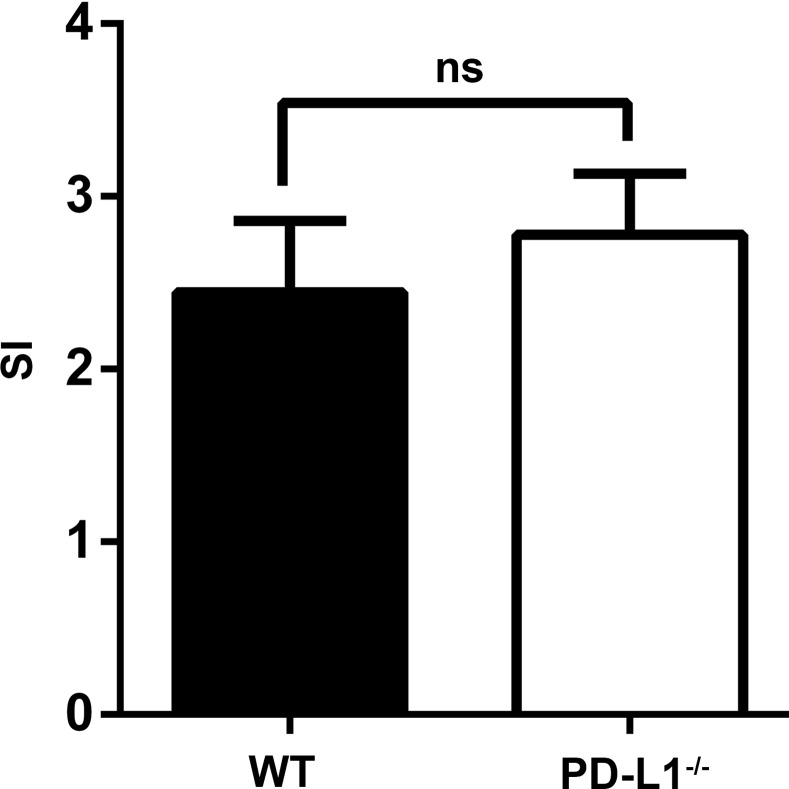
Influence of PD-L1 deficiency on insulin secretion in islets during glucose stimulation. Insulin release was measured using the insulin enzyme-linked immunosorbent assay kit, and the SI was calculated as the ratio of insulin release under high and low glucose conditions. Experiments were performed in triplicate, and the results represent three independent experiments. There was no difference in insulin secretion between the islets from WT and PDL1- deficient mice (ns indicates no significance).

The stimulation index (SI) was defined as the ratio of insulin levels that were released into the culture medium under high or low glucose conditions [20]. This simple, easy procedure is a useful parameter for the assessment of islet function. Here, to determine whether there was a difference in the function of insulin secretion between islets isolated from PD-L1^-/-^ and WT mice, glucose-stimulated insulin secretion was examined. The insulin levels increased approximately 3-fold in response to high glucose stimulation in islets from both groups (Fig 1). The SI was not significantly different between two groups (P = 0.35). Collectively, there was no difference in insulin secretion function between the islets from PD-L1^-/-^ and WT mice. Hence, PD-L1 deficiency had no detrimental effect on islet function.

### PD-L1 deficiency in grafted islets accelerated acute rejection

**Fig 2:**
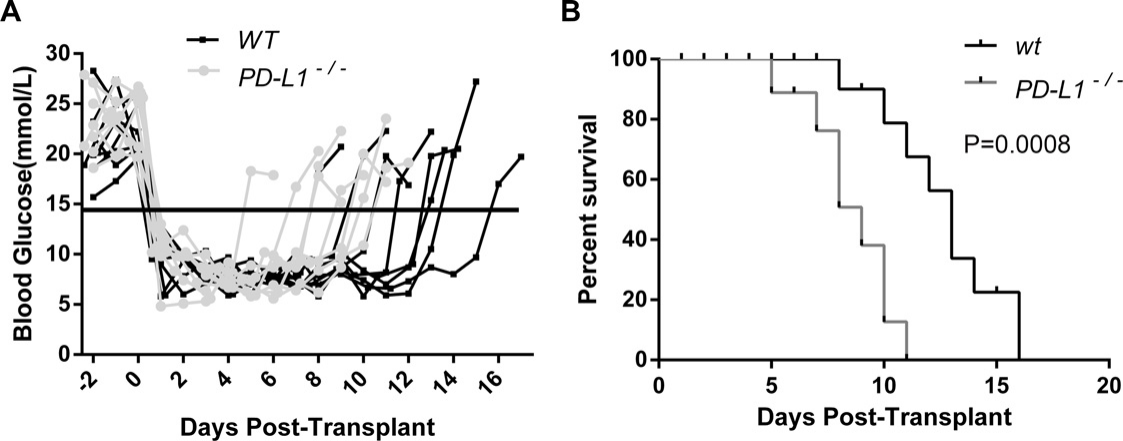
Survival of islet allografts from the donors of WT mice and PD L1-deficient mice. Blood glucose levels and islet graft survival in the recipients bearing islets from WT mice and PDL1- deficient mice are shown (N = 8). Diabetes was induced in BALB/c mice with STZ, followed by transplantation of four hundred islets isolated from WT or PDL1- deficient mice. The blood glucose levels are shown in Fig 2A in comparison with the glucose levels of the recipients transplanted with the islets from WT mice. Euglycemia was maintained for a shorter duration in the recipients bearing islets from PD-L1-deficient mice (Fig 2B). Graft survival was calculated by the Kaplan-Meier method and compared using a log-rank test (P = 0.0008). The results show that the survival time of the recipients transplanted with islets from PD-L1-deficient mice was clearly shorter compared with that of the recipients bearing islets from WT mice.

Because PD-L1 is involved in inducing immune tolerance during organ transplantation and weakening the inflammatory response, we hypothesized that PD-L1-deficient islets may advance immunity mediated allograft rejection. To verify this hypothesis, islets from PD-L1^-/-^ and WT mice were transplanted into the renal subcapsular space of diabetic BALB/c mice (a stable model of mouse islet allotransplant). As shown in Fig 2A, the implantation of 400 islets promptly reversed the blood glucose levels in STZ-induced diabetic recipients. Apparently, the blood glucose levels of recipients bearing of PDL1- deficient islets returned to hyperglycemia more promptly than those of the WT group. PD-L1 deficiency shortened the median graft survival time (MST) of transplanted islets (to 9 days) from the PDL1- deficient group compared with the WT group (to 13 days) (n = 8; P<0.001; Fig 2B). The PDL1- deficient islets were rejected significantly earlier.

### PD-L1 deficiency in grafted islets favored immune cell infiltration and decreased the islet function (insulin secretion)

**Fig 3:**
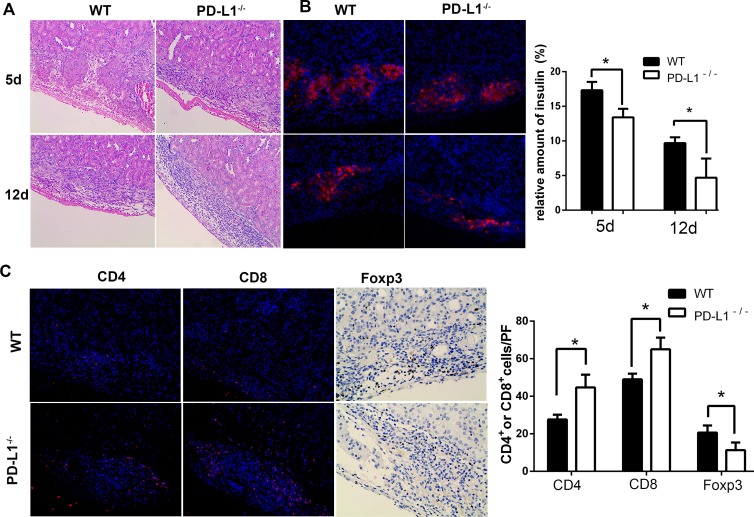
The residual β cells and infiltration of inflammatory cells at 5 and 12 days post-transplantation. Representative images of allografts in the renal capsules are shown (A) Grafts were harvested and stained for insulin with H&E. (B) Immunofluorescence staining for insulin (in red) in islet grafts. The right panel shows the relative quantification of the insulin signal analysed with Image-Pro Plus 6.0. Less insulin secretion was observed in the PD-L1-deficient group at both 5 and 12 days post-transplantation. (C) Immunofluorescence staining for inflammatory cells (CD4^+^ and CD8^+^, in red; Foxp3^+^, in brown) in islet grafts. Nuclei were counterstained with DAPI (in blue). Magnification is 200× (* P<0.05).

To explore the mechanism through which PD-L1 deficiency reduced the islet allograft survival time in diabetic mice, the grafts were dissociated at day 5 and day 12 post-transplantation and processed for histological analysis. We observed fewer residual islets from Fig 3A and decreased insulin secretion at both 5 and 12 days post-transplantation in the PD-L1-deficient islet grafts compared with the control group. Furthermore, at 12 days post-transplantation, the insulin positive mass was almost absent in PD-L1-deficient islets grafted beneath the renal capsule, as shown in Fig 3(B). Simultaneously, immunofluorescence analysis revealed that more CD4^+^ and CD8^+^ cells infiltrated the islet grafts in the PDL1-deficient group than those in the WT group at 5 days post-transplantation (Fig 3C; CD4^+^ cells, 49±3 in PD-L1-deficient group vs. 27.7±2.5 in WT group, CD8^+^ cells, 65±6.2 in PD-L1-deficient group vs. 44.7±6.8 in WT group per 200x field, three randomly selected fields were counted, P<0.05). However, there were fewer Foxp3^+^ T cells in the islet grafts in PD-L1-deficient group than those in WT group (P<0.05).

### PD-L1 deficiency in grafted islets enhances recipient alloreactivity

**Fig 4:**
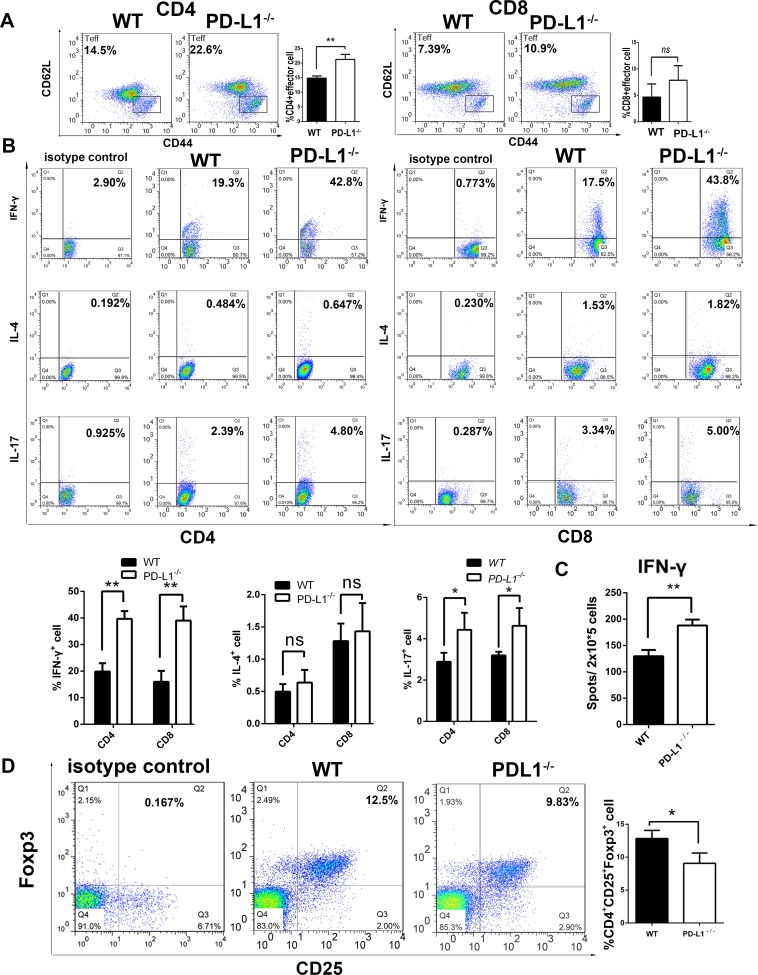
Analysis of effector T-cell, Treg and alloreactive cytokine-producing T cell generation. (A) Flow cytometry analysis of effector CD4^+^ and CD8^+^ T T-cell frequency. CD4^+^ and CD8^+^ effector T cells were identified by their staining characteristics (CD4^+^ CD44^high^ CD62^low^ and CD8^+^ CD44^high^ CD62^low^). The number of CD4^+^ effector T cells was significantly increased in the splenocytes harvested from recipients that had been transplanted with PDL1- deficient islet allografts. However, the number of CD8^+^ effector T-cells was not of a significant difference between the two groups. (B) Frequency of alloreactive cytokine-producing cells in splenocytes from recipients transplanted with islet allografts from WT and PDL1-deficient mice. Splenocytes were harvested 5 days after transplantation and were challenged with irradiated donor stimulators. The frequencies of IFN-γ, IL-4 and IL-17–producing CD4^+^T and CD8^+^T cells were then determined by flow cytometry. (C) The frequency of alloreactive T cells producing IFN-γ were detected by ELISpot assay. The recipients that received islets from PD-L1^-/-^ mice produced a significant increased amount of IFN-γ. (D) The frequency of Tregs (Foxp3^+^ T cells) was determined by flow cytometry, and there was a significant difference between the two groups. The data represent the means±SD of triplicate results from three mice per group (ns indicates no significance, * P<0.05, **P<0.01).

First, we examined the percentage of overall CD4^+^ and CD8^+^T cells in the recipients’ spleen and the results were shown as follows: CD4^+^ cells, 49.9±2.46% in PD-L1-deficient group vs. 41.7±1.87% in WT group (P<0.05), CD8^+^ cells, 15.53±2.25% in PD-L1-deficient group vs. 14.23±1.3% in WT group (P>0.05). Then, we compared the frequency of effector CD4^+^ and CD8^+^ T cells (CD62 low, CD44 high phenotype) among the splenocytes of both recipient groups. As shown in Fig 4A, the number of CD4^+^ effector T cells were higher in the recipients transplanted with PDL1- deficient islets (21.23±1.72%, P = 0.0041) than in the WT group (14.83±0.76%). However, no difference was present in the frequency of CD8^+^ effector T cells in the PDL1- deficient group (7.84±2.78%) versus WT (4.63±2.52%, P = 0.2113).

Moreover, we measured the frequency of alloreactive T cells producing IFN-γ, IL-4 and IL-17 in a mixed-leukocyte reaction assay. Splenocytes from recipients were harvested 5 days after transplantation and were then challenged with irradiated donor splenocytes. Flow cytometry was performed to quantify the number of alloreactive T cells producing IFN-γ, IL-4 and IL-17. The splenocytes from recipient mice transplanted with PDL1- deficient islets exhibited a significantly greater number of alloreactive cells producing IFN-γ and IL-17 in both the CD4^+^ and CD8^+^ T cell populations compared with the mice implanted with WT islets. We also observed slightly more IL-4-producing cells in the PD-L1-deficient group (Fig 4B). To confirm the flow cytometry results, the frequency of IFN-γ-producing cells was detected through an ELISpot assay. Expectedly, the recipients implanted with islets from PD-L1^-/-^ mice produced a significantly higher amount of IFN-γ (P<0.01; Fig 4C). Furthermore, we detected the frequency of Foxp3^+^ T cells (Tregs) among the splenocytes obtained from the recipients. Fewer Foxp3^+^ T cells were detected in the splenocytes from recipient transplanted with PDL1-deficient islets compared with the control group. Thus, PDL1-deficient donor islets enhance the recipient alloimmune responses by activating CD4^+^ T and CD8^+^ T ccells.

## Discussion

T1DM, an autoimmune disease, is characterized by insulin-dependence. Although several therapeutic approaches for T1DM have been developed, islet transplantation exhibits many advantages. Islet transplantation may make patients independent to external insulin, allowing them to not the pain associated with insulin injections, reducing hypoglycemia and the risks of end-organ damage, and improving QoL. Furthermore, islet transplantation is helpful for decreasing ocular, diabetic, renal, neurologic, and cardiovascular complications induced by T1DM[21]. Thus, islet transplantation is becoming the most attractive therapy.

However, allograft rejection seriously affects graft survival. It has been reported that many factors influence islet rejection. P2X7R, one of the lymphocytic ionotropic purinergic P2X receptors, may promote islet rejection through accelerating T cell activation. P2X7R antagonism has been shown to synergize with rapamycin in inducing islet allograft tolerance[22]. Dendritic cells (DCs), which are the dominant antigen-presenting cells, are also very important for T cell activation. Paolo Fiorina[23] reported that DCs from donor islets accelerate allograft rejection through a process related to the high expression of the chemokine receptors CCR5, CXCR4, CCR7, and CD86 and major histocompatibility complex class II in DCs. The depletion of DCs within donor islet prior to transplantation results in a significantly prolonged islet allograft survival. The B7-CD28 co-stimulatory pathway greatly affects the immune response of T cells. Most importantly, the signaling pathways promote the activation of T cells and may also attenuate T cell responses. ICOS-B7h, a novel pathway of the B7-CD28 family, also influences immune rejection and tolerance mediated by T cells. Mohammed Javeed I. Ansari et al [24] reported that ICOS blockade prolongs islet allograft survival in C57BL/6 recipients but is ineffective in reversing diabetes in NOD mice transplanted with islets. Recently, it has been reported by Moufida Ben Nasr[25] that co-transplantation of autologous MSCs prolonged islet allograft survival and promoted revascularization of transplanted islets through generating a local immunoprivileged sit by abrogating Th17 response and up-regulating IL-10^+^ regulatory T cells. In addition, there are many strategies for promoting immune tolerance and islet allograft survival, such as the induction of chimerism with hematopoietic stem cells (HSCs), the use of an anti-CD3-specific antibody, costimulation blockade, the targeting of dendritic cells, anti-TNF-α administration and the induction of Tregs [26]. PD-L1 and PD-L2, ligands for PD-1, are members of the negative co-stimulating molecules (B7 homologues). Similar to PD-L2, PD-L1 is not strongly expressed in resting cells. PD-L1 is up-regulated in activated B, T, myeloid, and dendritic cells. Moreover, PD-L1 is also expressed on many non-hematopoietic tissues, such as heart, placenta, pancreas, brain, muscle, and endothelium [16, 27–29]. Many studies have demonstrated that PD-L1 favors allograft survival. The synergistic activity of PD-L1.Ig plus CsA and PD-L1.Ig plus RPM leads to long-term cardiac allograft survival [14]. Additionally, Giuseppe Vassalli et al demonstrated that localized PD-L1.Ig expression in donor hearts attenuates acute allograft rejection in a rat model [30]. Furthermore, in recent years, Yang et al [16] first demonstrated that PD-L1 deficiency within a donor heart resulted in earlier rejection and more serious vasculopathy in a mouse chronic rejection model with enhanced recipient T cell alloreactivity. However, PDL1-deficient recipients did not exhibit accelerated cardiac allograft rejection. Even more interesting is the observation that PD-L1 expression on cardiac tissue alone significantly prolonged graft survival compared with full PDL1-deficient donor grafts in transplanted wild-type recipients using chimeric animals as donors. Paolo Fiorina recently reported that islet engraftment is promoted through the targeting of the CXCR4-CXL12 axis by mobilized autologous hematopoietic stem cells (HSCs), which do not express costimulatory molecules (such as CD80, CD86, and CD40) but highly express PD-L1[15]. Several studies have demonstrated that recipients treated with PD-L1.lg exhibit prolonged graft islet survival. Moreover, β-cell-targeted PD-L1- CTLA4Ig overexpression reduced allo-islet rejection [31, 32]. The porcine peripheral blood B cell line L23 with targeted PD-L1 overexpression reduced cellular and antibody responses to xenografts [33]. Consistent with the reports above, in our study, the survival time of PDL1-deficient grafted islets was significantly reduced compared with the WT group (MST 9 days in the PDL1-deficient-islet group vs. MST 13 days in the WT islet group, p<0.001, n = 8).

To explore the underlying mechanisms, the preservation of islets and amount of inflammatory cell infiltration was measured by histology. The number of residual insulin-positive cells was significantly decreased at 5 days and 12 days post-transplantation in the recipients transplanted with PDL1-deficient islets compared with the islets from the WT mice. PD-L1 is involved in CD4^+^ and CD8^+^ T cell proliferation and is essential for the induction of regulatory cells [34–38]. Mohamed H. Sayegh reported that PD-L1 blockage accelerates CD4^+^ T cell-mediated skin allograft rejection [39]. In this study, we observed more CD4^+^ and CD8^+^ T cells in the PDL1-deficient allograft compared with the WT islet group. However, there were fewer Treg cells were found in the PDL1-deficient allograft. In the mixed-lymphocyte reaction assay, we observed an increased number of IFN-γ and IL-17–producing alloreactive T cells in the PD-L1 deficient allograft group. In addition, the ELISpot assay results revealed more IFN-γ spots in the PD-L1-deficient allograft group. Our results are well in line with those reported by Keir et al [40] who demonstrated that islet cell expression of PD-L1 inhibits pathogenic self-reactive CD4^+^ T cell–mediated tissue destruction in autoimmune diabetes by limiting IL-2 and IFN-γ production during naive CD4^+^ T cell activation in a non-obese diabetic mouse model. Moreover, we observed more CD4^+^ effector T cells among the splenocytes from the PD-L1 deficient group than among those from the WT group, which was consistent with the report by Yang [16] that PD-L1 deficiency in the donor heart enhanced allograft rejection in a major histocompatibility complex class II–mismatched model of vascularized cardiac allograft rejection. Although PD-L1 deficiency in the donor heart does not influence allograft rejection, PD-L1-deficient islets enhanced allograft rejection. Thus, the expression of PD-L1 is of greater importance in islet transplantation. Taken together, the results show that PD-L1 deficiency within islet allografts promotes alloreactive T cell activation, inflammatory cell infiltration and allograft rejection. However, the detailed mechanism underlying these effects remains to be determined.

Collectively, this study is the first report indicating that PD-L1 deficiency within an islet allograft reduces mouse islet allotransplant survival. We also confirmed the importance of islet allograft PD-L1 expression regarding immune tolerance. Thus, the desire to explore a better protocol to make β-cells that over-express PD-L1 for transplantation in the diabetic recipient instead of the common islet reducing immune therapy for the treatment of T1DM may come true.
